# Chemical Composition and Monoterpenoid Enantiomeric Distribution of the Essential Oils from Apharsemon (*Commiphora gileadensis*)

**DOI:** 10.3390/medicines4030066

**Published:** 2017-09-12

**Authors:** Nativ Dudai, Alona Shachter, Prabodh Satyal, William N. Setzer

**Affiliations:** 1 Agricultural Research Organization, Unit of Medicinal and Aromatic Plants, Newe Ya’ar Research Center, POB 1021, Ramat Yishay 30095, Israel; nativdud@gmail.com (N.D.); alonash@agri.gov.il (A.S.); 2 Department of Chemistry, University of Alabama in Huntsville, Huntsville, AL 35899, USA; prabodhsatyal@gmail.com; 3 Aromatic Plant Research Center, 615 St. George Square Court, Suite 300, Winston-Salem, NC 27103, USA

**Keywords:** Balm of Judea, Burseraceae, traditional medicine, essential oil composition, chiral gas chromatography

## Abstract

**Background:** *Commiphora gileadensis* (Hebrew: apharsemon) has been used since Biblical times to treat various ailments, and is used today in the traditional medicine of some Middle Eastern cultures. **Methods:** The essential oils from the stem bark, leaves, and fruits of *Commiphora gileadensis*—collected at the Ein Gedi Botanical Garden, Israel—were obtained by hydrodistillation and analyzed by gas chromatography–mass spectrometry. In addition, the enantiomeric distributions of the monoterpenoids in the essential oils have been determined by chiral gas chromatography. **Results:** The essential oils were dominated by monoterpene hydrocarbons, followed by oxygenated monoterpenoids. The major components in *C. gileadensis* oils were the monoterpenes α-pinene (11.1–18.4%), sabinene (15.8–35.9%), β-pinene (5.8–18.0%), *p*-cymene (4.8–8.4%), limonene (1.3–6.2%), γ-terpinene (0.7–8.1%), and terpinen-4-ol (5.3–18.5%). The (–)-enantiomers predominated for α-pinene, sabinene, β-pinene, limonene, and terpinen-4-ol. **Conclusions:** The chemical compositions of the *C. gileadensis* essential oils from Israel are markedly different from previously reported samples, which were rich in sesquiterpenoids. Likewise, the enantiomeric distribution of monoterpenoids is very different from *Boswellia* spp. essential oils.

## 1. Introduction

*Commiphora gileadensis* (L.) C. Chr. (syn. *C. opobalsamum* (L.) Engl.) is an medium-sized aromatic shrub native to the Red Sea area, including Saudi Arabia, Yemen, Oman, Eritrea, Ethiopia, Egypt, and Kenya [1]. In 1763, the renowned scholar and botanist Carl Linnaeus received a specimen collected in Yemen from his student Peter Forsskål, and identified it as the Biblical “Balm of Gilead” [2,3,4]. Today, there is widespread agreement among scholars that *C. gileadensis* is one of the myrrh species of the Burseraceae and the source of the legendary “Balm of Judea” (“Balsam”) perfume.

*C. gileadensis* is also known in Hebrew as “apharsemon” (5). The plant is indigenous to the land of Israel and emits a thick aromatic sap, from which the renowned perfume was made. Various historical sources point out that apharsemon was cultivated intensively in the Jordan Valley and Ein Gedi, in the western Dead Sea basin as a crop, and its perfume was admired and traded at a high price during Biblical times, during the time of the Second Jewish Temple, and during the Roman and Byzantine eras [5,6,7]. Archeological excavations around the Ein Gedi oasis in Israel, on the north-west shore of the Dead Sea, unraveled furnace-like structures, jars, and other objects dating from the sixth century B.C.E. that were comparable with those found in perfume-making workshops. This supports the historical references. In the 1990s, *C. gileadensis* was reintroduced to Israel and is currently cultivated in Ein Gedi, near the Dead Sea [8], renewing a long lost part of Jewish history in the land of Israel.

*C. gileadensis* has been used historically to treat a wide array of ailments [9,10,11,12,13], and is used today in the traditional medicine practices of some cultures in the Middle East. In Yemen and Oman, the bark exudate is used externally to treat skin disorders such as burns, wounds, and infections [6,14,15]. Among the Arab populations in the Middle East, a decoction of the aerial parts of the plant is administered as a pain reliever, a diuretic, and a laxative [16,17]. In 2010, Iluz et al. [6] investigated the medicinal properties of *C. gileadensis* and demonstrated that its sap had an inhibitory effect against *Bacillus cereus* and was capable of blocking lectins of *Pseudomonas aeruginosa*. This confirms the historical sources that indicated the antiseptic properties of the balsam’s sap. In 2012, Amiel et al. [18] further supported this finding by showing that (*E*)-caryophyllene was a key component in *C. gileadensis* essential oil, that the balsam’s stem extracts acted against cancerous tumor cells, and had apoptosis-inducing properties that acted selectively, eradicating tumor cells, but not healthy cells.

Although *C. gileadensis* has been an important medicinal plant throughout history, information about the essential oil composition of wild *C. gileadensis* is lacking. Therefore, in this work, the essential oil compositions attained from the stem bark, leaves, and fruits of wild *C. gileadensis* collected in Ein Gedi, Israel were determined and compared to the composition of commercially used *C. gileadensis* oil. In addition, to further characterize the essential oils, the enantiomeric distributions of the monoterpenoids in *C. gileadensis* essential oils were determined.

## 2. Materials and Methods

### 2.1. Plant Material

Leaves, stems, and fruits of *C. gileadensis* were collected at the Ein Gedi Botanical Garden. The plant was identified and collected by Nativ Dudai. Plant parts were placed within a Clevenger-type apparatus for 1.5 h and the essential oil was hydro-distilled. The commercial samples were produced by a steam distillation apparatus and supplied by the grower Mr. Guy Erlich from his plantation at Almog (near the Dead Sea), Israel.

### 2.2. Gas Chromatography–Mass Spectrometry

The essential oil compositions of wild and commercial *C. gileadensis* were analyzed by GC-MS using a Shimadzu GCMS-QP2010 Ultra (Shimadzu Scientific Instruments, Columbia, MD, USA) operated in the electron impact (EI) mode (electron energy = 70 eV) with a scan range of 40–400 amu and a scan rate of 3.0 scans/s, and GC-MS solution software. The GC column was a ZB-5 fused silica capillary column (Phenomenex, Torrance, CA, USA), 30 m length × 0.25 mm inner diameter, with a (5% phenyl)-polymethylsiloxane stationary phase and a film thickness of 0.25 μm. The carrier gas was helium, with a column head pressure of 552 kPa and flow rate of 1.37 mL/min. Injector temperature was 250 °C and the ion source temperature was 200 °C. The GC oven temperature program was set for an initial temperature of 50 °C and increased at a rate of 2 °C/min to 260 °C. A 5% *w*/*v* solution of the sample in CH_2_Cl_2_ was prepared, and 0.1 µL was injected with a splitting mode of 30:1. Identification of the oil components was based on retention indices determined by reference to a homologous series of *n*-alkanes, and by comparison of their mass spectral fragmentation patterns with those reported in the literature [19] and stored in our in-house MS library. Percentages were determined based on total ion current integrations of the peak areas, and are uncorrected.

### 2.3. Chiral Gas Chromatography–Mass Spectrometry

Chiral analysis of the essential oils of wild and commercial *C. gileadensis* was performed on a Shimadzu GCMS-QP2010S (Shimadzu Scientific Instruments, Columbia, MD, USA) operated in the EI mode (electron energy = 70 eV) with a scan range of 40–400 amu and a scan rate of 3.0 scans/s The GC was equipped with a Restek B-Dex 325 capillary column (Restek Corp., Bellefonte, PA, USA) (30 m × 0.25 mm ID × 0.25 μm film). The oven temperature began at 50 °C, and was then gradually raised to 120 °C at a rate of 1.5 °C/min. After reaching 120 °C, the oven temperature was increased to 200 °C at 2 °C/min intervals and kept at this temperature for 5 min. Helium was the carrier gas, and the flow rate was maintained at 1.8 mL/min. Samples were diluted to 3% *w*/*v* with CH_2_Cl_2_, and a 0.1 µL sample was injected in a split mode at a split ratio of 1:45. The monoterpenoid enantiomers were identified by comparison of retention times with authentic samples obtained from Sigma-Aldrich (Milwaukee, WI, USA). Relative enantiomer percentages were determined based on peak areas.

## 3. Results and Discussion

**Figure 1:**
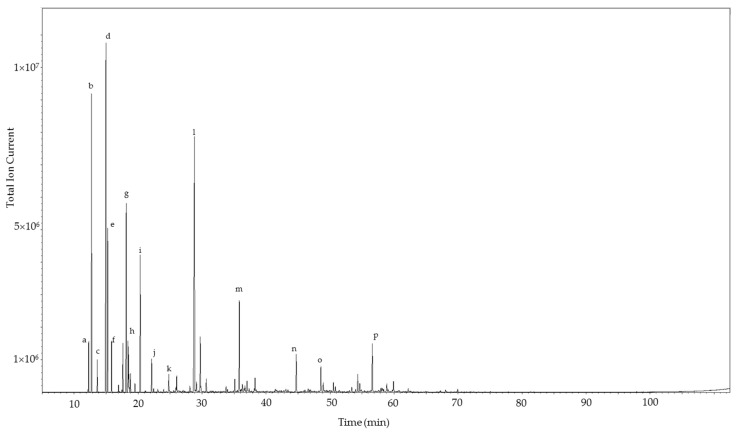
Gas chromatogram of the leaf essential oil of *Commiphora gileadensis*. The major components are indicated: a, α-thujene; b, α-pinene; c, camphene; d, sabinene; e, β-pinene; f, myrcene; g, *p*-cymene; h, limonene; i, γ-terpinene; j, terpinolene; k, *cis-p-*menth-2-en-1-ol; l, terpinen-4-ol; m, bornyl acetate; n, (*E*)-caryophyllene; o, germacrene D; p, 1,10-di-*epi*-cubenol.

The essential oil compositions of *C. gileadensis* are compiled in Table 1. The essential oils were dominated by monoterpene hydrocarbons, followed by oxygenated monoterpenoids. The major components in *C. gileadensis* oils were the monoterpenes α-pinene (11.1–18.4%), sabinene (15.8–35.9%), β-pinene (5.8–18.0%), *p*-cymene (4.8–8.4%), limonene (1.3–6.2%), γ-terpinene (0.7–8.1%), and terpinen-4-ol (5.3–18.5%). Sesquiterpenoid concentrations were very low in these samples, and constituted only 0.8%, 10.6%, and 2.4% in the stem, leaf, and fruit oils, respectively. A representative gas chromatogram is shown in Figure 1.

A previous examination of *C. gileadensis* oils from aerial parts and flowering tops collected in Makkah, Saudi Arabia showed a complete absence of monoterpene hydrocarbons, but there were high concentrations of terpinen-4-ol (8.5% and 9.8%), and of sesquiterpenoids: δ-cadinene (5.0% and 4.8%), α-calacorene (9.4% and 3.8%), viridiflorol (4.6% and 4.4%), τ-muurolol (4.5% and 3.5%), and cadalene (4.3% and 5.4%) [20]. Another investigation of *C. gileadensis* oil from leaves and fruits from Ein Gedi, Israel showed high concentrations of α-pinene (7.2%) and sabinene (21.1%), and richness in sesquiterpenes (*E*)-caryophyllene (20.1%) and germacrene D (19.6%) [18]. The high concentrations of sesquiterpenes in these earlier studies are in marked contrast to the *C. gileadensis* oils examined in the present study, suggesting a wide variation of essential oil compositions within the population of this plant species.

A preponderance of sesquiterpenoids over monoterpenoids in members of *Commiphora* has been previously shown for *C. kua* [21] and *C. habessinica* [22], where monoterpenoids have not been detected); and for *C. parvifolia* [23], where monoterpene hydrocarbons and oxygenated monoterpenoids were present at a level of 16%. On the other hand, *C. tenuis* from Ethiopia was rich in monoterpene hydrocarbons with 60.8% α-pinene, 8.8% β-pinene, 8.9% α-thujene, 6.3% sabinene, and 5.5% limonene, but had only trace amounts of sesquiterpenoids [24].

*Commiphora leptophloeos* leaf essential oil had nearly equal concentrations of monoterpenes and sesquiterpenes with α-phellandrene (26.3%), β-phellandrene (12.9%), (*E*)-caryophyllene (18.0%), α-humulene (5.5%), and germacrene D (5.6%) predominating [25]. In contrast, *C. ornifolia* essential oil showed no monoterpene hydrocarbons and only 4.7% sesquiterpene hydrocarbons; the bulk of the essential oil composition was made up of oxygenated monoterpenoids (56.3%) and oxygenated sesquiterpenoids (16.4%) [23].

Although the *C. gileadensis* essential oils were dominated by monoterpenoids, several of these have demonstrated antimicrobial activity, validating the use of exudates from this plant to treat burns, wounds, and infections. Thus, for example, α-pinene [26,27], sabinene [28], β-pinene [29], *p*-cymene [30,31], limonene [29], γ-terpinene [32], and terpinen-4-ol [32,33] have shown antimicrobial activities.

**Figure 2:**
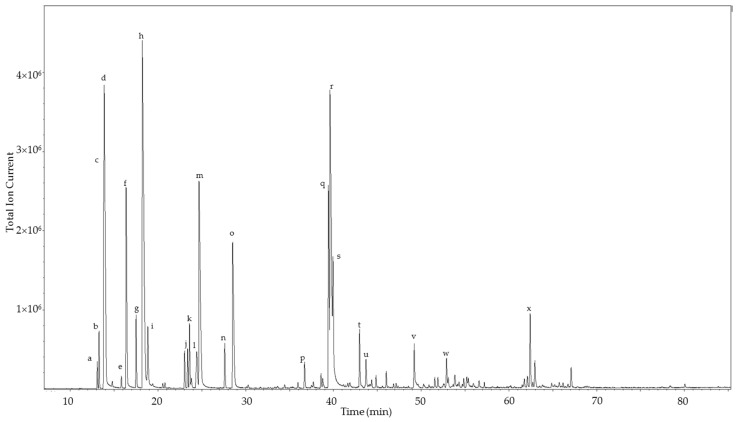
Chiral gas chromatogram of the leaf essential oil of *Commiphora gileadensis*. The major components are indicated: a, (+)-α-thujene; b, (−)-α-thujene; c, (+)-α-pinene; d, (−)-α-pinene; e, (+)-β-pinene; f, (−)-β-pinene; g, (+)-sabinene; h, (−)-sabinene; i, myrcene; j, (+)-limonene; k, α-terpinene; l, (−)-limonene; m, *p*-cymene; n, terpinolene; o, γ-terpinene; p, (−)-borneol; q, (+)-terpinen-4-ol; r, (−)-terpinen-4-ol; s, (−)-bornyl acetate; t, (−)-α-terpineol; u, (+)-α-terpineol; v, (*E*)-caryophyllene; w, germacrene D; x, 1,10-di-*epi*-cubenol.

The enantiomeric distributions of the monoterpenoids in *C. gileadensis* essential oils are summarized in Table 2. The (−)-enantiomers predominated in α-thujene, α-pinene, sabinene, β-pinene, limonene, terpinen-4-ol, and α-terpineol. Borneol and bornyl acetate showed exclusively the (−)-enantiomers, but (+)-linalool and (+)-linalyl acetate predominated over the (−)-enantiomers. The chiral gas chromatogram of the leaf oil is shown in Figure 2. Currently, there is little information about the enantiomeric distribution of terpenoids in members of the Burseraceae, and apparently none regarding *Commiphora* spp. However, *Boswellia* species have been examined (Table 2) [34,35,36]. *Boswellia carterii* essential oil from Somalia also showed predominantly the (−)-enantiomers in α-thujene, α-pinene, sabinene, β-pinene, and limonene [35]. Basar and co-workers found that (+)-α-thujene dominates in *B. carterii* from Ethiopia [34]. In contrast, the (+)-enantiomers dominated in α-thujene, α-pinene, sabinene, and β-pinene in *B. sacra* essential oil from Oman [35]. On the other hand, analysis of a *Boswellia* sp. from Somalia showed (−)-α-thujene, (+)-α-pinene, (+)-sabinene, (+)-limonene, and (+)-terpinen-4-ol predominating [36]. (−)-Bornyl acetate was observed in the *B. carterii* sample from Ethiopia [34].

Chiral gas chromatography has been used as an analytical technique to detect outliers from authenticated oils and the possible adulteration of commercially important essential oils [37]. Since *C. gileadensis* has the potential for commercial utilization, this study serves to establish the enantiomeric distributions of the monoterpenoids in authentic unadulterated *C. gileadensis* essential oils. In addition, this work provides a comparison between *C. gileadensis* and other members of the Burseraceae (*Boswellia* spp.).

## 4. Conclusions

The chemical composition of essential oils from the stems, leaves, and fruits of *Commiphora gileadensis* have been shown to be rich in monoterpenoids, but a dearth of sesquiterpenoids, in contrast to previous reports of *C. gileadensis* essential oils; there is apparently wide variation in *C. gileadensis* volatiles. The enantiomeric distribution of monoterpenoids showed little variation between the different essential oil samples of *C. gileadensis*, contrasted markedly from *Boswellia* spp. essential oils.

## Figures and Tables

**Table 1:** Chemical compositions of the essential oils of wild and commercial *Commiphora gileadensis*.

RI ^a^	Compounds	Wild Plant Parts			Commercial
		Stem	Leaf	Fruit	Oil
890	Styrene	0.1			0.1
900	Nonane			0.1	
921	Tricyclene	0.1	0.1	0.1	0.1
924	α-Thujene	2.8	1.6	1.6	1.7
932	α-Pinene	15.4	11.2	15.9	18.4
946	α-Fenchene	tr ^b^		tr	
948	Camphene	1.4	1.0	0.7	1.5
952	Thuja-2,4(10)-diene				0.1
964	6-Methylheptan-2-ol	tr			0.1
972	Sabinene	28.5	15.8	35.9	29.1
977	β-Pinene	7.5	5.8	18.0	8.2
988	Myrcene	2.8	1.7	2.2	1.7
1002	Octanal			0.1	
1003	*p*-Mentha-1(7),8-diene (Pseudolimonene)			tr	
1006	α-Phellandrene	0.4	0.2	tr	0.2
1008	δ-3-Carene	tr			
1014	1,4-Cineole		0.1		
1016	α-Terpinene	3.0	1.8	0.1	2.0
1024	*p*-Cymene	4.8	8.4	5.3	6.4
1028	Limonene	1.9	1.9	6.2	1.3
1029	β-Phellandrene	1.6	1.4	1.3	1.0
1031	1,8-Cineole	0.2	0.2		0.2
1034	(*Z*)-β-Ocimene	0.6	0.7	0.3	0.8
1044	(*E*)-β-Ocimene	0.1	0.3	tr	0.1
1057	γ-Terpinene	8.1	5.8	0.7	5.9
1068	*cis*-Sabinene hydrate	0.1	0.1	0.1	0.2
1084	Terpinolene	1.2	1.3	0.2	0.7
1088	*p*-Cymenene	tr	0.1	tr	0.1
1098	α-Pinene oxide			0.1	
1098	Linalool	0.1	0.1		1.0
1099	*trans*-Sabinene hydrate	tr		0.1	0.1
1103	Nonanal			tr	
1112	2-Methyl-6-methylen-octa-1,7-dien-3-one		0.1		
1123	*cis-p*-Menth-2-en-1-ol	0.7	0.8	0.2	0.2
1124	Cyclooctanone	0.1	0.1	tr	tr
1126	*allo*-Ocimene	tr			tr
1127	4,5-Epoxy-*trans*-carene			tr	
1134	Terpin-3-en-1-ol	tr	0.1		
1139	*trans*-Pinocarveol	0.1	0.2	0.1	0.5
1141	*trans-p*-Menth-2-en-1-ol	0.4	0.7	0.2	0.2
1143	*trans*-Verbenol	0.1		tr	0.1
1145	Camphor		0.1		
1155	Sabina ketone	tr	0.1	0.1	0.1
1160	Pinocarvone			tr	0.1
1167	α-Phellandrene epoxide			tr	tr
1170	Borneol	0.1	0.3	0.1	0.4
1180	Terpinen-4-ol	11.6	18.5	5.3	6.9
1182	Thuj-3-en-10-al				tr
1183	*p*-Methylacetophenone	tr			tr
1185	*p*-Cymen-8-ol	0.1	0.4	0.2	0.2
1190	Myrtenol		tr	tr	tr
1193	α-Terpineol	1.3	2.6	0.7	0.9
1195	*cis*-Piperitol	0.2	0.2	0.1	
1204	Decanal			0.6	
1206	Verbenone				0.1
1207	*trans*-Piperitol	0.3	0.6	0.1	0.1
1216	*endo*-Fenchyl acetate	tr			tr
1217	*trans*-Carveol				tr
1223	Nerol				tr
1241	Cuminaldehyde				0.1
1248	Linalyl acetate	tr			4.4
1255	Methyl citronellate	0.1	0.1		0.1
1270	1-Decanol			tr	
1278	*cis*-Linalool oxide acetate (pyranoid)	tr	0.1		tr
1282	Bornyl acetate	2.9	4.4	0.4	3.3
1285	Isobornyl acetate	tr	0.1	tr	0.1
1287	Thymol	tr	0.1		
1291	*p*-Menth-1-en-9-ol			tr	
1294	Terpinen-4-ol acetate	tr			0.1
1295	Carvacrol	0.1	0.3		tr
1300	Tridecene			tr	
1317	*p*-Mentha-1,4,-dien-7-ol			0.1	
1321	*p*-Menth-8-en-1,7-diol			0.1	
1321	Methyl decanoate	0.1	0.1		tr
1339	*p*-Cymene-8-ol acetate			0.1	
1345	α-Terpinyl acetate				0.1
1349	Eugenol	tr			
1356	Neryl acetate	tr			0.1
1360	Decanoic acid			tr	
1367	α-Ylangene		0.2		
1374	α-Copaene				tr
1376	Geranyl acetate				0.1
1381	α-Bourbonene		0.1		
1397	Methyleugenol	0.1	0.1		
1407	Dodecanal			0.1	
1417	(*E*)-Caryophyllene	0.1	1.9	0.1	tr
1439	6,9-Guaiadiene		0.1		
1447	*trans*-Muurola-3,5-diene		0.2		
1453	α-Humulene	tr	0.1	tr	
1457	Alloaromadendrene			0.1	tr
1476	γ-Curcumene	0.1			
1479	Germacrene D	0.1	1.2	0.2	0.1
1489	Viridiflorene	tr	0.1		
1493	Bicyclogermacrene	tr	0.1		
1496	α-Muurolene		0.1	0.1	tr
1500	*epi*-Zonarene	tr	0.1		
1502	(*E*,*E*)-α-Farnesene		0.1		tr
1505	β-Bisabolene	tr	0.1		0.1
1511	γ-Cadinene	tr	0.4	tr	
1516	δ-Cadinene	0.1	0.3	0.3	tr
1526	α-Panasinsen		0.1		
1559	(*E*)-Nerolidol		0.2		
1569	(3*E*)-Hex-3-enyl benzoate		0.1		
1574	Spathulenol		0.9	0.3	
1580	Caryophyllene oxide		0.5	tr	tr
1583	Globulol		0.2		
1592	Viridiflorol		tr		
1613	1,10-di-*epi*-Cubenol	0.2	2.6		tr
1630	*iso*-Spathulenol		0.1		
1637	Alloaromadendrene epoxide		0.2	0.1	
1640	α-Muurolol (Torreyol)	tr	0.2	0.1	
1642	τ-Muurolol		0.1	0.2	
1644	δ-Cadinol		0.1	0.1	
1653	α-Cadinol	tr	0.4	0.8	
1657	Eudesm-4(15),7-dien-1α-ol		0.1	tr	
1666	β-Atlantone	tr			
1688	Eudesma-4(15),7-dien-1β-ol		0.1		
1730	Oplopanone			0.1	
1860	Platambin		0.1	tr	
	Monoterpene hydrocarbons	80.2	59.0	88.6	79.4
	Oxygenated monoterpenoids	18.6	30.0	8.0	19.5
	Sesquiterpene hydrocarbons	0.5	4.9	0.7	0.3
	Oxygenated sesquiterpenoids	0.3	5.7	1.7	tr
	Others	0.3	0.4	1.0	0.2
	Total Identified	99.9	100.0	100.0	99.4

^a^ RI = Retention index determined with respect to a homologous series of *n*-alkanes on a ZB-5 column. ^b^ tr = trace (<0.05%).

**Table 2:** Enantiomeric distribution [(+):(−)] of monoterpenoids from *Commiphora gileadensis* and *Boswellia* species essential oils.

Compounds	*C. gileadensis*				*B. sacra* [35]	*B. carterii* [35]	*Boswellia* sp. [36]	
	Stem	Leaf	Fruit	Commercial				
α-Thujene	30:70	29:71	28:72	28:72	73.5:26.5	3.8:96.2	43:57	36:64
α-Pinene	25:75	28:72	29:71	28:72	89.2:10.8	40.5:59.5	83:17	76:34
Sabinene	8:92	8:92	8:92	7:93	87.5:12.5	14.5:85.5	93:7	98:2
β-Pinene	3.5:96.5	3:97	3.7:96.3	3:97	75.2:24.8	40.8:59.2		
Limonene	33:67	32:68	32:68	31.6:68.4	21.3:78.7	27.0:73.0	73:27	65:35
Linalool	59:41	59:41		60:40				
Borneol	0:100	0:100	0:100	0.1:99.9				
Terpinen-4-ol	30:70	31:69	30:70	30:70			81:19	71:29
α-Terpineol	30:70	32:68	30:70	31:69				
Linalyl acetate				99:1				
Bornyl acetate	0:100	0:100	0:100	0:100				
